# Disturbing the rhythm of thought: Speech pausing patterns in schizophrenia, with and without formal thought disorder

**DOI:** 10.1371/journal.pone.0217404

**Published:** 2019-05-31

**Authors:** Derya Çokal, Vitor Zimmerer, Douglas Turkington, Nicol Ferrier, Rosemary Varley, Stuart Watson, Wolfram Hinzen

**Affiliations:** 1 School of Engineering and Computer Science, Queen Mary University of London, London, United Kingdom; 2 Department of Language and Cognition, University College London, London, United Kingdom; 3 Institute of Neuroscience, Newcastle University, Newcastle upon Tyne, United Kingdom; 4 Northumberland Tyne and Wear NHS Foundation Trust, Newcastle, United Kingdom; 5 ICREA (Institució Catalana de Recerca i Estudis Avançats), Barcelona, Spain; 6 Department of Translation and Language Sciences, Universitat Pompeu Fabra, Barcelona, Spain; 7 FIDMAG Germanes Hospitalaries Research Foundation, Benito Menni Hospital, Barcelona, Spain; Consejo Nacional de Investigaciones Cientificas y Tecnicas, ARGENTINA

## Abstract

Everyday speech is produced with an intricate timing pattern and rhythm. Speech units follow each other with short interleaving pauses, which can be either bridged by fillers (*erm*, *ah*) or empty. Through their syntactic positions, pauses connect to the thoughts expressed. We investigated whether disturbances of thought in schizophrenia are manifest in patterns at this level of linguistic organization, whether these are seen in first degree relatives (FDR) and how specific they are to formal thought disorder (FTD). Spontaneous speech from 15 participants without FTD (SZ-FTD), 15 with FTD (SZ+FTD), 15 FDRs and 15 neurotypical controls (NC) was obtained from a comic strip retelling task and rated for pauses subclassified by syntactic position and duration. SZ-FTD produced significantly more unfilled pauses than NC in utterance-initial positions and before embedded clauses. Unfilled pauses occurring *within* clausal units did not distinguish any groups. SZ-FTD also differed from SZ+FTD in producing significantly more pauses before embedded clauses. SZ+FTD differed from NC and FDR only in producing longer utterance-initial pauses. FDRs produced significantly fewer fillers than NC. Results reveal that the temporal organization of speech is an important window on disturbances of the thought process and how these relate to language.

## Introduction

Spontaneous speech in people with schizophrenia has long been shown to manifest altered patterns of linguistic organization, including reduced syntactic complexity and increased syntactic errors [1–4]. This is unsurprising given close links between thought and language: speech production is nothing other than the process of converting a thought into a temporal sequence of speech units. Disturbances of the thought process are therefore likely to be reflected in speech disturbances. This is obvious in the case of the symptom of formal thought disorder (FTD), which is manifest and diagnosed as disorganized speech [5] and hence closely related to language. Clinically, however, such speech is rated through terms such as derailment, illogicality, tangentiality, or poverty of content, and it is unclear what distinguishes it in linguistic terms. Recent work suggests that language in FTD can be differentiated from that of people with schizophrenia without FTD and controls by linguistically fine-grained variables, e.g. their anomalous use or production of definite noun phrases (e.g. *the man on the left*) but not indefinite ones (e.g. *a man who likes toast*) [6]. The same linguistic variables can distinguish FDRs from neurotypical controls [7]. People with FTD also differ from people with schizophrenia without FTD through a lower proportion of subordinated clauses (e.g. *Paul said* *that he liked it*), but not of syntactic errors [7]. Here we hypothesized that thinking disturbances may be reflected in the temporal organization of speech as well, i.e. the normal rhythm of structural linguistic units following each other as separated by intermittent pauses.

Pauses lasting between 250–3,000ms are an inherent feature of the normal speech process and cognitive functioning, making up a major part of speech time [8]. While this appears to be a universal feature of normal speech, some data (see [9]) suggest that pause durations can differ even across typologically and culturally closely-related languages, such as Spanish and Italian. Pauses have been interpreted as intermittent feedback or delay loops, in which the momentum of the semi-automatic speech generation process is halted, while the next unit of information is planned [10]. Such speech planning operations are sensitive to the structural organization of speech (i.e., what type of speech unit is to be generated next). Of particular importance is the clausal unit, i.e. subject-verb phrase complexes like *he ran*. Pauses occurring *within* such clausal units have often been related to speech planning at the level of lexical retrieval or concepts, [8, 11–14] while pauses at clausal boundaries are more related to planning at the level of the content of the subsequent utterance, i.e., the thought to be expressed, [11, 12, 15, 16] and are more likely if this (subsequent) utterance is more syntactically complex [17]. Apart from silent or empty (‘unfilled’) pauses, dysfluencies can be marked through fillers such as *erm*, *ah*, *um* or *er*, which bridge a speech gap. Fillers have been related functionally to speech monitoring [18] and hearer-oriented social signaling, [19] particularly in utterance-initial positions [20].

Units of linguistic organization map onto the kind of thought expressed–i.e. the units of thought. Thus, clausal boundaries enclose units of speech that map onto complete thoughts, e.g. *[she dressed herself]*, while non-clausal units do not, e.g. *[herself which]*. An altered speech rhythm can thus reflect and illuminate disturbances of the thought process. People with schizophrenia have been found to differ from neurotypical controls in terms of a greater number of pauses, proportion of silence, total length of pauses, and mean pause duration, in a reading task [21]. Patients with undifferentiated schizophrenia and with FTD also manifest a smaller proportion of fillers as compared to controls [20, 22]; no differences were found when distinguishing pauses by whether they occurred within or between utterances [22]. Since some parameters in this domain are automatically extractable from speech recordings and objectively quantifiable, several studies have highlighted their potential role as biomarkers [21, 23]. Since different types of pauses serve different cognitive functions, [16, 18, 24] pausing patterns could also be a useful comparative window into cognitive dysfunction across different pathologies [5, 19, 25–31]. In schizophrenia, the significance of language for SZ extends beyond FTD and could help understanding the etiology of other symptoms as well [32–34]. However, the paucity of studies, small sample sizes (e.g. N = 6), differences in variable definitions across studies, [35] lack of fine-grained differentiation of pauses by syntactic position, [2, 20–22] lack of differentiation of short and long pause types, and lack of group comparisons of patients with and without FTD, [2, 21, 22] prevent clinically useful generalizations at this point.

### Aims of this study

We aimed to clarify dysfluency patterns that may mark spontaneous speech in schizophrenia (SZ). We recruited 15 participants with (SZ+FTD) and 15 without (SZ-FTD) formal thought disorder, 15 first-degree relatives (FDR) and 15 neurotypical controls (NC). We hypothesized that the schizophrenia groups would be different in their dysfluency patterns from the non-clinical groups, that the former would differ from each other, and that the two non-clinical groups would differ from each other as well. We predicted that the SZ groups would be more dysfluent than the non-clinical groups, and that they would be more dysfluent when a clausal or utterance boundary is involved. That is, more pauses of either short or long durations before embedded clauses and before utterances, but not within clauses (i.e., when the pause is followed by a non-clausal unit), would be produced by the SZ groups. Since fillers have often been related to social signaling, we predicted fewer fillers would be produced by SZ groups relative to non-clinical groups. As linguistic measures are often related to general cognitive measures in SZ, and patients with FTD are marked by greater cognitive decline than patients without FTD, [36–38] we also explored associations between dysfluency patterns (i.e., unfilled pauses and fillers) and general cognition (IQ), age, and education.

## Methods

### Participants

A favourable ethics opinion for this study was obtained from National Research Ethics Service Committee North East—Newcastle & North Tyneside 2. All participants provided written and informed consent. Fifteen participants with FTD (SZ+FTD) and fifteen without (SZ-FTD) were recruited from a UK secondary care mental healthcare trust (Northumberland, Tyne and Wear (NTW) NHS Foundation Trust) (Table A in S1 File for psychotropic medication). All participants with SZ met DSM-IV diagnostic criteria and scored at least 60 on the Positive and Negative Symptom Scale for Schizophrenia (PANSS), which was selected as the cut-off point to generate a sample in which all participants were symptomatic at least at the level found in stable outpatients. Trained and experienced raters completed PANSS; inter-rater reliability checks revealed internal consistency ratings.

In addition, we placed adverts in university and hospital buildings to recruit fifteen NC participants. Fifteen FDRs of people with schizophrenia were recruited via NTW Trust carer and patients’ groups. For neurotypical control participants and FDRs, exclusion criteria included past or current psychotic disorders; for all participants, exclusion criteria included: substance dependence or abuse, pervasive developmental disorders interfering with language skills, severe epilepsy, significant head injury, stroke, or brain tumor. We did not formally check whether any participants were professionally trained in diction but had no evidence that this was the case. All subjects were native speakers of English.

Participants with SZ were dichotomised based upon their score on PANSS question 2 (‘Conceptual Disorganisation’, CD). Participants who scored = > 4 (which equates to an anchor of at least ‘moderate’ on this item) were categorised as SZ+FTD, those who scored = < 3 (‘minimal’) were categorised as SZ-FTD. Eleven of the fifteen FDRs who completed the PANNS scored one on the CD scale (= no conceptual disorganization), while three scored two (= questionable pathology) and one scored three (= circumstantial, tangential or paralogical thinking, difficulty in directing thoughts towards a goal, some loosening of associations evidenced under pressure).

The SZ-FTD participants were significantly younger than SZ+FTD participants (p = .009) (Table 1), and also significantly less educated than NC and FDR groups (p = .024; p = .016). However, the two clinical groups’ educational levels were not significantly different (*p* > .05). Question 2 on the PANSS was our measure of FTD and did not correlate with total length of illness (rho = .056). In addition, there was no correlation between total length of illness and the relevant PANSS sub-scale (positive symptoms (rho = -.046) or with PANSS total score (rho = -.097) in either the FTD sub-group, or the combined group of patients with schizophrenia (using Spearman’s [*p* < 2.8, p > .15]).

**Table 1:** Socio-demographic features of groups.

Group	Sex #male	Age	Years of education	IQ	Comparisons	Age		Education		IQ/WASI	
		Mean (s.d.)	Mean (s.d.)	Mean (s.d.)		t (df)	^1^p	t (df)	p	t (df)	p
NC	7	45 (13)	16 (4)	107 (8.4)	NC vs. SZ+ FTD	.952 (28)	>.05	1.142 (28)	> .05	-5.763* (28)	.001
FDR	8	45 (13)	17 (4)	103 (10)	NC vs. SZ-FTD	1.836 (28)	>.05	2.386* (28)	.024	3.584* (28)	.001
SZ-FTD	13	38 (7)	13 (4)	86 (20)	NC vs. FDR	.042 (28)	>.05	-.330 (28)	> .05	1.151 (28)	>.05
SZ+ FTD	10	50 (15)	15 (4)	80 (16)	FDR vs. SZ+ FTD	.952 (28)	>.05	1.403 (28)	> .05	-4.738* (28)	.001
					FDR vs. SZ-FTD	1.787 (28)	.085	-2.568* (28)	.016	-2.815* (28)	.009
					SZ+ FTD vs. SZ-FTD	-2.825* (28)	.009	-1.324 (28)	> .05	1.008 (28)	>.05

Mean (M) and Standard Deviation (s.d.), and independent t-test comparisons of age, education, and WASI IQ for neurotypical controls (NC), first degree relatives (FDR), and participants with (SZ+FTD) and without formal thought disorder (SZ-FTD)

^1^Sig. (2‐tailed)

* The mean difference is significant at the .05 level.

While SZ-FTD and SZ+FTD were matched on WASI IQ score (WASI-II) (*p* >.05), both clinical groups had significantly lower IQ scores than NC and FDR.

### Linguistic testing

All participants were instructed to describe an eight-picture comic strip in which a cat steals a fish intended for dinner guests [39]. The interviewer visited participants either at home or in clinical units. NCs and FDRs visited the Institute of Neuroscience at Newcastle University for interviews.

All audio files were anonymized as to participant and diagnosis. Each file was transcribed by three native speakers of English and then, using the F4 transcript program (https://www.audiotranskription.de/english/f4), tagged for unfilled pauses and fillers. Codings were agreed upon via a consensus method by four native speakers of English.

Subsequently, each sentence was digitized at a 10 kHz rate and analyzed with an F4 waveform editor. Unfilled pauses were measured from the offset of activity associated with the preceding utterance or word to the start of the subsequent utterance or word. Following cut-offs similar to those used in previous studies, we excluded very short pauses less than 250ms, [20–22] and pauses longer than 3seconds [14, 21, 22]. We did this by adopting the common assumption that pauses less than 250ms are phonatory gaps linked to the respiratory cycle, [8, 21, 22] which are very hard to discriminate [9]. Regarding pauses longer than 3s, there is some evidence that pauses longer than 2s tend to be rare in neurotypical speech, [9] and in our data pauses longer than 3s tended to signal that a participant was no longer engaged with the given task. The percentage of excluded pauses >3secs in our data amounted to 4% of the total number of pauses. To also explore possible group differences in pause length, we introduced a further categorical distinction between unfilled pauses lasting 250-1000ms (‘short’) and 1000-3000ms (‘long’). There is evidence that people with schizophrenia are distinguishable from neurotypical controls particularly through longer mean pause durations [21]. Consistent with previous studies on sentence dysfluency in patient groups, we did not code the duration of filled pauses. Such durations depend on complex issues such as the choice of filler and the relation between fillers and prolongations [2], which we did not investigate here.

Since the study’s first author was never in contact with participants and blind to diagnoses, she checked and annotated all participants’ dysfluency patterns regarding the annotation scheme described below. During the annotation process, agreement on all questionable cases was achieved by raters.

### Annotation and speech dysfluency variables

For this study, the following linguistic definitions were adopted:

- Utterance—a grammatically independent unit of discourse providing new information.
- Clause–a configuration with a subject and predicate (usually a verb).
- Embedded clause–a clause that forms a syntactic constituent of another clause.

In addition, speech dysfunction was captured by three main dysfluency patterns: First, syntactic positions of unfilled pauses were subdivided and tagged into three categories: (1) utterance-initial pauses–silent/unfilled pauses prior to an utterance: (2) within-clause pauses followed by non-clausal units; and (3) pauses before embedded clauses–unfilled pauses before a speaker attaches an embedded clause to the main clause. Second, two types of durations of unfilled pauses (less< 1 sec. [short] vs. between 1–3 secs. [long]) were annotated. Third, fillers were tagged according to the same three syntactic positions (Table B in S1 File for examples).

### Data analysis

Statistical analysis involved comparison of unfilled pauses and fillers across groups, followed by a comparison of unfilled pauses indexed by syntactic position, by duration, and by fillers indexed by syntactic position. To control for the overall quantity of dysfluencies, the number of unfilled pauses and fillers in every individual participant was converted into ratios. The denominator was chosen depending on whether the variable of interest made direct reference to a particular unit of speech, such as utterances, clauses, or embedded clauses. Thus, whether participants in one group produce more unfilled pauses in utterance-initial positions naturally depends on how many utterances they produce. E.g., the following ratios were thus used: (a) total number of utterance-initial unfilled pauses/total utterances, (b) within-clause pauses/total clauses, and (c) unfilled pauses before embedded clauses/total embedded clauses (see Table C in S1 File for all ratios).

Since general cognition (IQ), age, and education varied across groups, we ran covariance analyses for two main variables (unfilled pauses/total utterances & fillers/total utterances) to explore the effect of group on the production of unfilled pauses/fillers controlling for the effects of IQ, age, and education. For unfilled pauses/total utterances, which were normally-distributed, an analysis of covariance (ANCOVA) with Bonferroni *post hoc* test was conducted. For fillers/total utterances, a non-normally distributed variable, log-transformations proved unfeasible. Therefore, we performed Quade’s rank analysis of covariance, [40] with the ranked dependent variable residualized over the covariants (i.e., IQ, age, and education). Subsequently, a one-way analysis of variance (ANOVA), using the residuals as dependent variable and the grouping as the factor, was conducted.

For the remaining variables, non-parametric tests, including Mann-Whitney U pairwise comparisons (when Kruskal-Wallis showed a significant effect of group) were used with Bonferroni correction. A Bonferroni correction was applied by dividing the alpha value by the number of group comparisons. The threshold was set at .008. Since the covariant analyses of unfilled/fillers did not reveal main effects of general cognition (IQ), age, and education, further covariant analyses for the remaining variables were not computed.

## Results

### Group comparisons for speech dysfluencies: unfilled pauses and fillers

The proportion of all dysfluency variables did not distinguish groups (see Figure A and Table C in S1 File). There was also no group effect on unfilled pauses when we added co-variants (IQ, age, and education) to the analysis (see Figure B and Table C in S1 File), Test of between subject effects: IQ effect on unfilled pauses: F(1, 59) = 2.038, p = .159, age effect on unfilled pauses F(1, 59) = .059 p = .808, education effect on unfilled pauses F(1, 59) = 3.016, p = .088 (see Table D S1 File).

**Fig 1:**
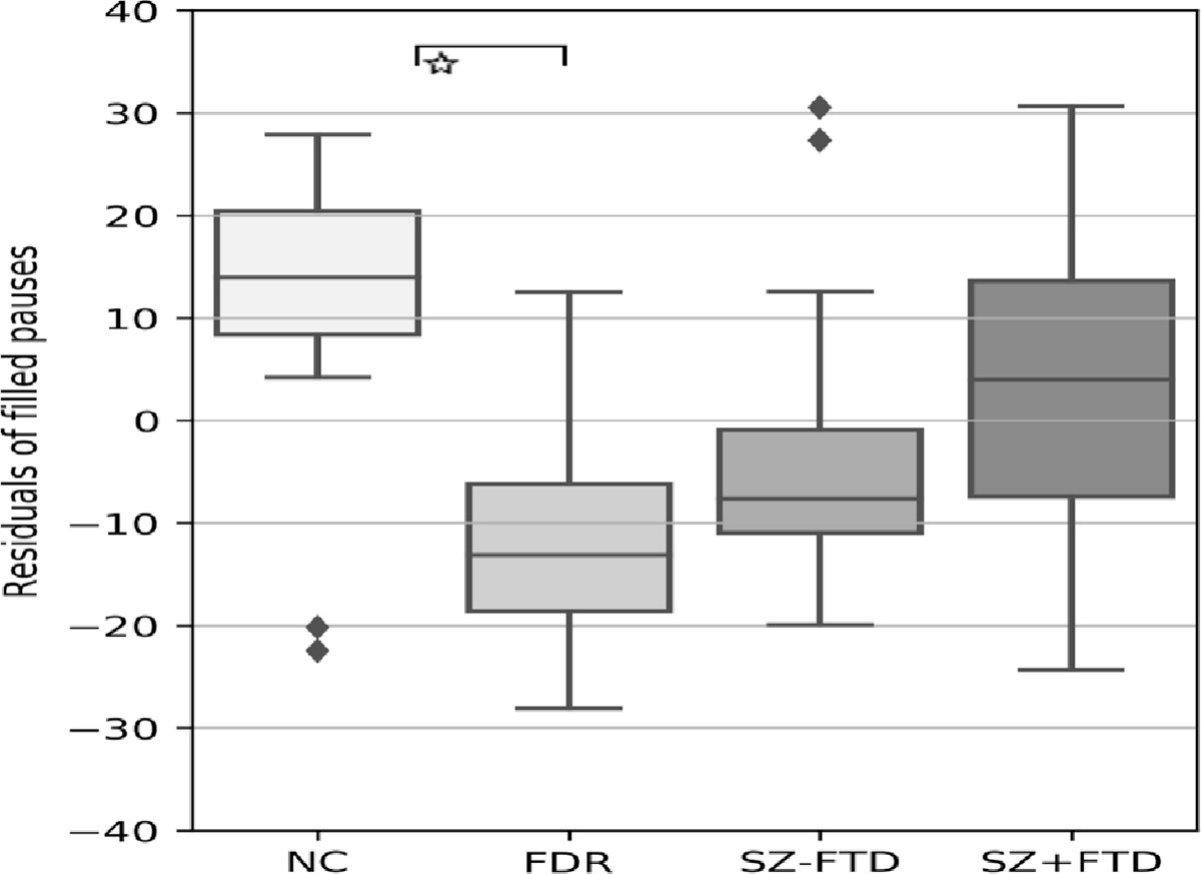
Mean of residualized filled pauses to total of utterances over the covariants (i.e., IQ, age, and education) across neurotypical controls (NC), first-degree relatives (FDR), participants with SZ+FTD and without thought disorder (SZ-FTD). Error bars show 95% confidence interval.

A rank analysis of covariance of fillers with IQ, age, and education as covariates revealed a main effect of group on the production of fillers. FDR produced significantly fewer fillers compared to NC (see Fig 1 and Table E in S1 File).

### Syntactic position of unfilled pauses

#### Utterance-initial unfilled pauses

**Fig 2:**
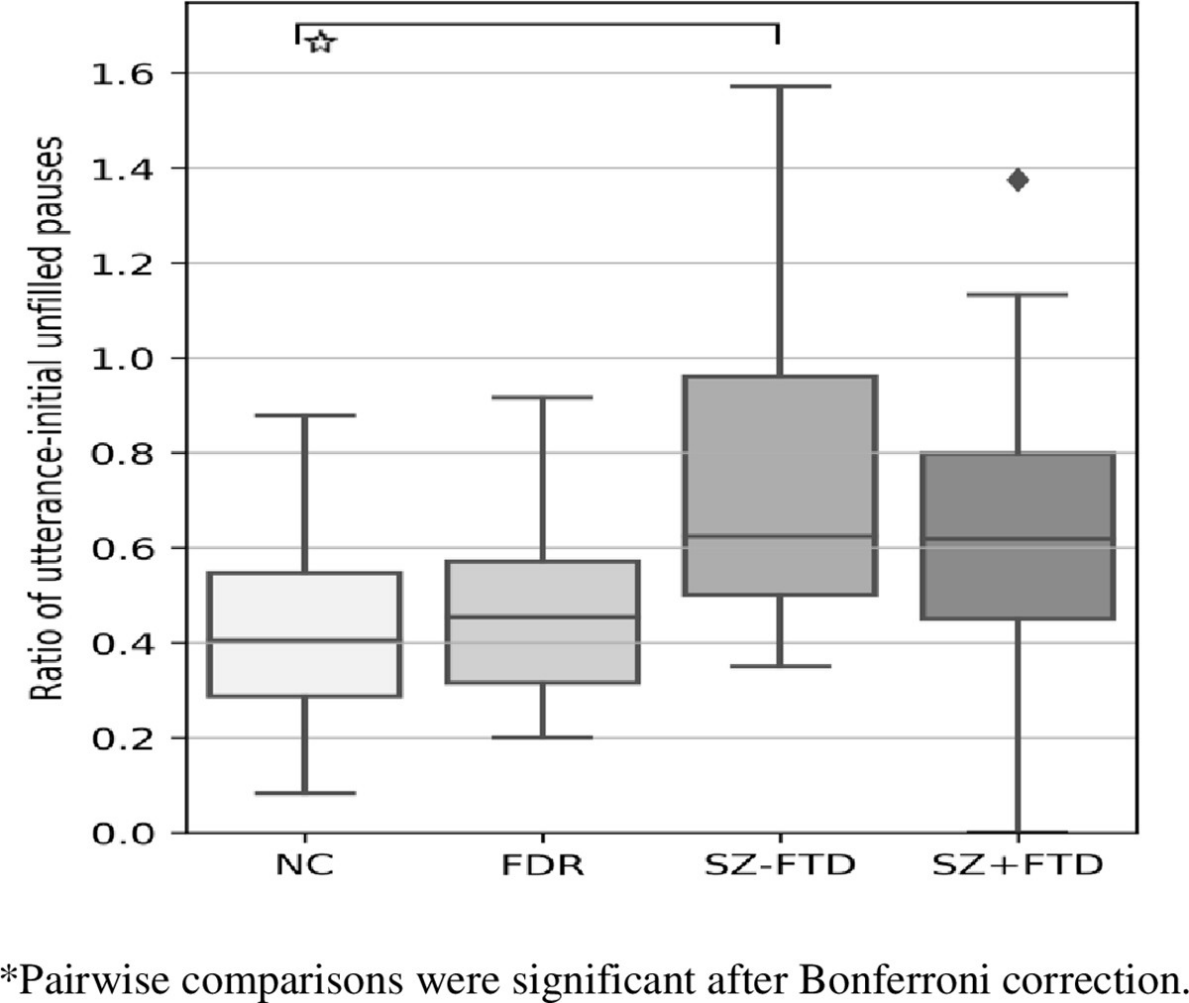
Ratio of total number of utterance-initial empty pauses to total utterances across neurotypical controls (NC), first-degree relatives (FDR), participants with SZ+FTD and without thought disorder (SZ-FTD). Error bars show 95% confidence interval.

The highest proportion of utterance-initial pauses occurred in the SZ-FTD group, while NC had significantly lower proportions in comparison (see Fig 2, Figure C and Tables C and F in S1 File).

#### Within-clause pauses

Kruskal-Wallis revealed no significant group effect for within-clause pauses, p >.05 (Tables C and G in S1 File).

#### Pauses before embedded clauses

**Fig 3:**
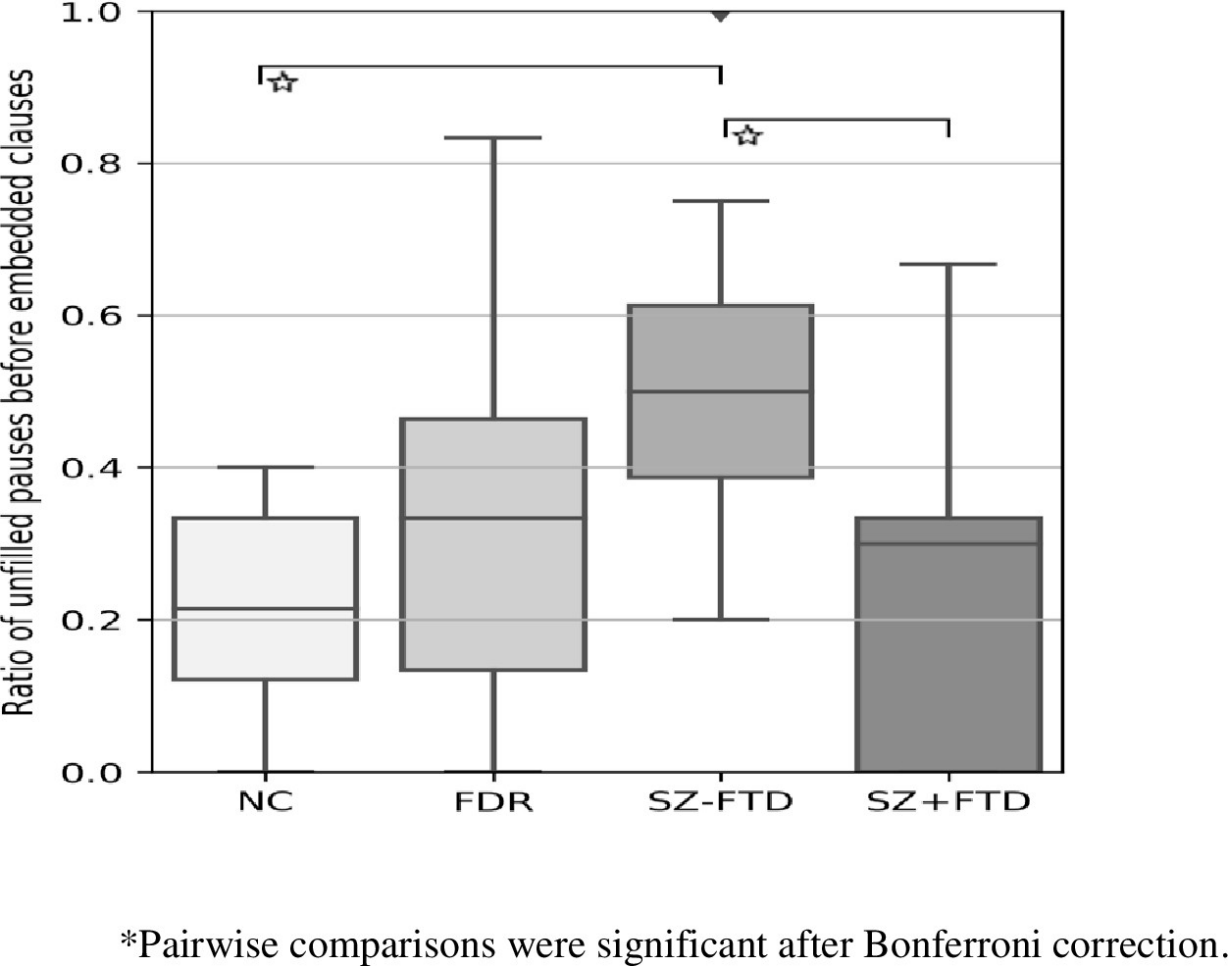
Ratio of total number of empty pauses before embedded clauses to total of embedded clauses across neurotypical controls (NC), first degree relatives (FDR), participants with (SZ+FTD) and without thought disorder (SZ-FTD). Error bars show 95% confidence interval.

There was a large and significant difference in the proportion of empty pauses before embedded clauses between SZ-FTD and NC. The two SZ groups also differed significantly in this respect (see Fig 3, Figure E and Tables C and H in S1 File.).

### Length of pauses

**Fig 4:**
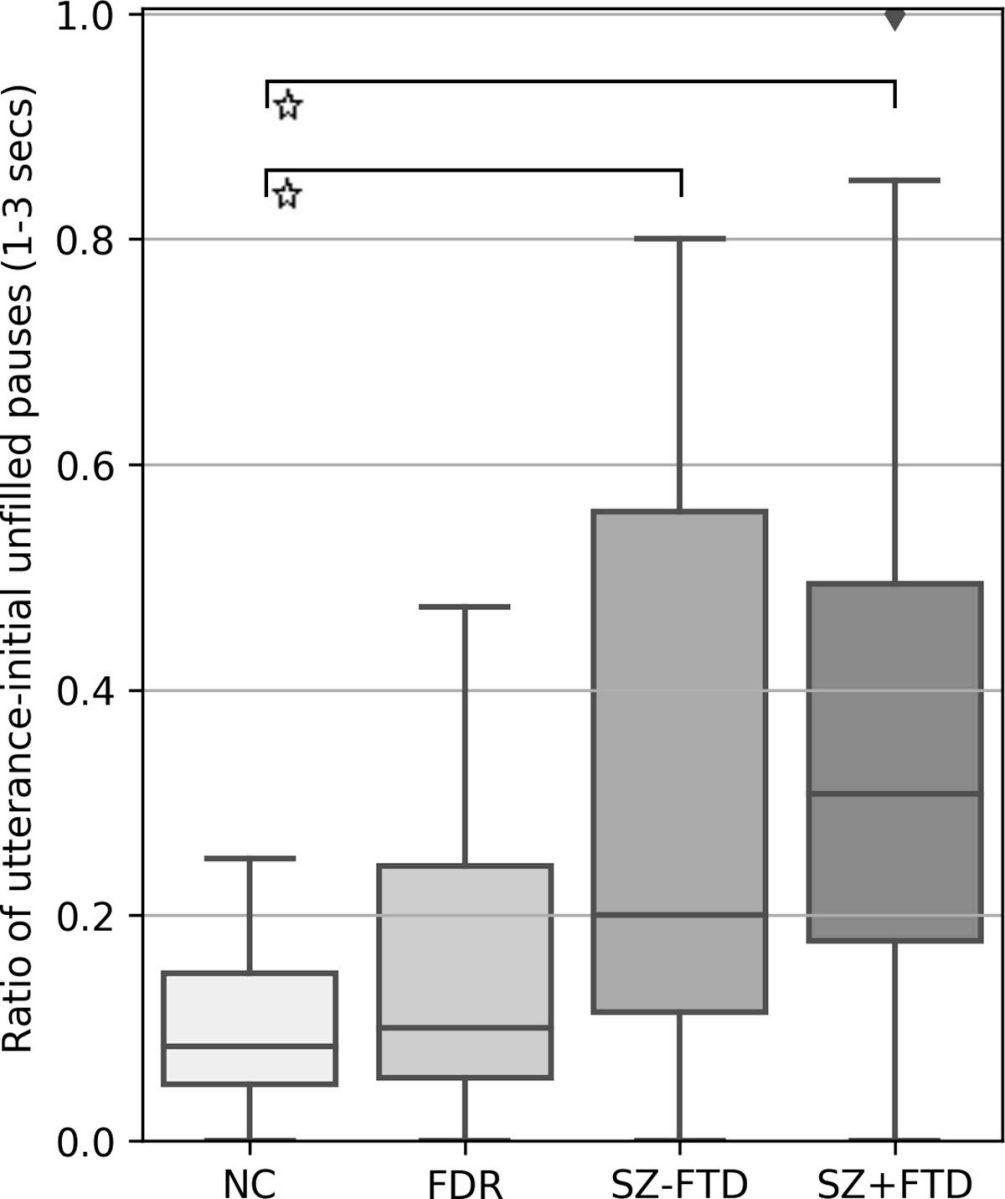
Ratio of total number of utterance-initial empty pauses between one and three seconds to total of utterances across neurotypical controls (NC), first-degree relatives (FDR), participants with (SZ+FTD) and without thought disorder (SZ-FTD). Error bars show 95% confidence interval.

While a Kruskal-Wallis test did not reveal a significant group effect for utterance-initial pauses shorter than one second, it did reveal one for utterance-initial pauses between 1–3 seconds, p = .004. In addition, Mann-Whitney U test comparisons with Bonferroni corrections showed that both SZ+FTD and SZ-FTD produced significantly more utterance-initial pauses between 1–3 secs than NC (see Fig 4 and Tables C and F in S1 File, as well as Figure C in S1 File for the histogram of length of utterance-initial pauses).

#### Within-clause pauses

Kruskal-Wallis revealed no significant group effect for within-clause pauses with regard to length of pauses, p >.05 (Tables C and G in S1 File; Figure D in S1 File for the histogram of length of within-clause pauses).

#### Length of pauses before embedded clauses

NC and SZ-FTD differed when the length of pauses before embedded clauses was taken into account (Tables C and H in S1 File) (see Figure E in S1 File for histogram of length of embedded clause pauses). Compared to NC, SZ-FTD had a higher ratio of pauses before embedded clauses of between 1–3 seconds.

### Syntactic positions of fillers

**Fig 5:**
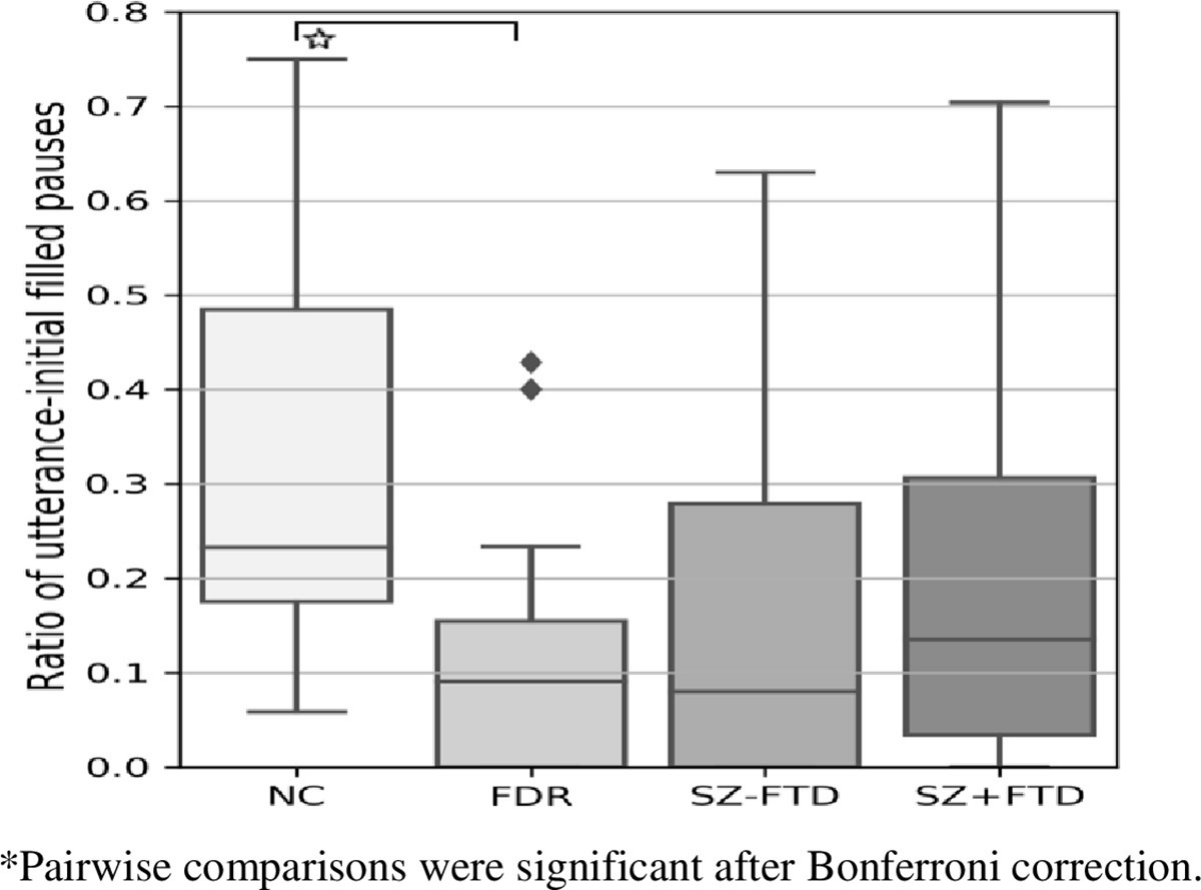
Ratio of utterance-initial fillers to total of utterances across neurotypical controls (NC), first-degree relatives (FDR), participants with SZ+FTD and without thought disorder (SZ-FTD). Median is 5. Because of lower margin, it is not seen.

FDR produced a significantly lower proportion of utterance-initial fillers than NC (Fig 5; Tables C and I in S1 File). Kruskal-Wallis did not reveal a significant group effect for within-clause fillers and fillers before embedded clauses (Tables C and J in S1 File, as well as Figure F in S1 File for the histogram of unfilled pauses).

## Discussion

These results confirm our hypothesis that dysfluency patterns distinguish participants with SZ from non-clinical controls, and both clinical and non-clinical groups from each other. However, differences between participants with and without SZ only started to appear when unfilled pauses and fillers were looked at separately and were indexed to specific syntactic positions. That is, unlike in earlier work, [21, 35] participants with SZ did not produce more unfilled (silent) pauses after co-varying for IQ, age, and education. However, those earlier results were obtained from reading tasks rather than spontaneous speech; nor did they discriminate between SZ with and without FTD. In the latter task, speakers must continuously generate new thoughts of their own. This may help to explain why an increase in empty pauses was found here only relative to specific syntactic positions, namely utterance-initial ones and those before embedded clauses. Clauses index units of information or thoughts of particular kinds. Strikingly, no group differences at all were found in within-clause pauses, contrasting with the alternative pattern seen in aphasia [27]. Within-clause pauses have been widely regarded as reflecting speech planning difficulties at the level of word-finding or lexical retrieval. This pattern suggests that in the speech generation process, the problem seen in SZ does not lie at a lexical but at a grammatical level: it arises at the boundaries of larger syntactic units where complete thoughts are encoded. Our findings specifically point to a difficulty in the organization of thought in speech at positions involving either the utterance or clausal boundaries in embedded positions. This finding contrasts with an earlier study [22], where no differences between a group of six people with FTD and neurotypical controls in unfilled pauses in clausal boundary positions was seen. However, these authors made no syntactic distinction between utterances and embedded clauses, no group with SZ but without FTD was included, and durations were not considered.

The pattern of empty pauses reported here raises three main questions: (i) what is the significance of the clause and utterance boundaries; and (ii) why this pattern is mostly confined to people with SZ *without* FTD (SZ-FTD) (namely, in utterance-initial pauses, pauses before embedded clauses, and in pauses before embedded clauses between 1-3s in length); and why, (iii), similarities appear between SZ+FTD and SZ-FTD only when the ‘long’ utterance-initial pauses (between 1 and 3 seconds) are considered. Taking these questions in turn, the clause is the smallest unit that maps onto a complete thought, in the sense that it can carry propositional information, for which minimally a subject and a predicate are required [41]. The results thus show that the disturbance at the level of thought clinically yielding a diagnosis of schizophrenia maps onto the syntactic boundary that demarcates thought-sized units, structurally containing the elements necessary to configure a complete thought. This suggests that the psychopathology in question somehow *involves* these elements and the configuration in which they occur. Regarding the second and third questions, the two key facts are that patients with FTD show a speech rhythm pattern distinguishable from that of neurotypical controls only in utterance-initial ‘long’ unfilled pauses and distinguishable from patients without FTD only before embedded clauses. This pattern can be summarized by saying that SZ+FTD shows sensitivity to the same syntactic positions, but differs from controls only when duration is considered, with differences showing only in the long utterance-initial pauses. Although this pattern depends on the pragmatic choice we made in distinguishing ‘short’ and ‘long’ pauses, with a cut-off at the 1s boundary, while not coding pauses less than 250ms and more than 3s, the pattern is intriguing. In response, we offer the following speculation. As noted, pausing in speech is an inherent element of mental health, sustaining a thought process as a previous utterance is moved out of the workspace and a new unit of thought is internally configured so as to be generated next. SZ-FTD thus appear to suffer from a disruption in this intermittent loop, with empty gaps (i.e., unfilled pauses) in the thought process appearing, in positions where such thoughts are to be generated for production. We suggest that SZ+FTD may suffer from the same impairment, since significant differences from neurotypical controls in unfilled pauses show at least partially in the same syntactic positions (namely utterance-initial ones). Yet, in traditional terms, patients with FTD also exhibit the symptom of ‘loosening of associations’: The latter may not allow this pattern of gaps to be seen in ‘short’ pauses, since an insufficiently controlled (‘loosened’) thought process leading to seemingly ‘unplanned’ and incoherent utterances may cover up such gaps, as long as they are short. Hence, while short gaps become undetectable, the thought disorder still shows in speech rhythmic patterns, yet now only when real gulfs are opening. That SZ+FTD significantly differed from SZ-FTD in pausing before embedded clauses may relate to the same loosening, as well as to embedded clauses being produced less by patients with FTD than without [7].

We also predicted that fillers would be produced less by patient groups than by non-clinical groups. Our results showed no evidence for this, insofar as neither differences between the SZ groups, nor between clinical and non-clinical groups reached significance after Bonferroni correction, unlike in previous studies of fillers in SZ [20, 22]. By contrast, FDRs strikingly differed in this respect from controls, but not from the clinical groups. NC not only produced more fillers than any other group, but the decrease in FDRs was again indexed by syntactic position, insofar as FDRs specifically produced significantly fewer fillers in utterance-initial positions. As noted in the introduction, fillers have often been related to inter-social signaling [20]. If so, it is tempting to suggest that this result may be due to the nature of the social interaction between FDRs and the people with SZ whose relatives they are. Unfortunately, information about living arrangements of our FDR participants, which could help to address this conjecture, was not available to us. In any case, this pattern suggests that empty gaps in speech are a more reliable indicator of thought disturbance than defects of social cognition as seen in fewer fillers.

Results of the covariant analyses suggest that the pattern of fillers as well as unfilled pauses (when indexed to syntactic positions) in our patient groups are not linked to IQ, age, or education, which is unlike in other domains of language, where such correlations have been found [7, 40]. They are thus linked to language in a more specific sense, raising intriguing questions about the role of speech generation networks in the neurocognitive basis of symptoms in schizophrenia and their potential clinical utility as biomarkers. Future work should also consider the potential effect of psychotropic medication on pause duration, which our study cannot exclude, as our sample was too small to assess the effects of individual drugs on the duration results. It should also address potential gender differences in the production of dysfluencies, since our study did not control for gender (see [42] for evidence for gender differences in the production of fillers); the same applies to cross-linguistic differences in pause durations, which could impact on results obtained in different languages (see [9]).

Overall, this study has laid new groundwork for investigating alterations of the thought process that defines schizophrenia at the level of speech rhythm, highlighting the significance of clausal units and the utterance boundary and thereby specific forms of grammatical complexity and meaning exhibited. The units in question and their temporal organization in speech are inherently linked to the normal thought generation process. Speech generation and timing provide a window into this process and an objective basis for tapping into how it can be disturbed.

## Supporting information

S1 File Supplementary materials. **Table A.** Number of neurotypical controls (NC), first degree relatives (FDR), and participants with (SZ+FTD) and without formal thought disorder (SZ-FTD) for psychotropic medication.
**Table B.** Speech dysfluency patterns.
**Table C.** Statistical significance for possible between-group differences in speech dysfluencies.
**Table D.** Pairwise comparisons of unfilled pauses/total utterances over covariants of IQ, age, and education by neurotypical controls (NC), first degree relatives (FDR), participants with FTD (SZ+FTD), and without FTD (SZ-FTD) in univariate analysis of variance.
**Table E.** Pairwise comparisons of residuals of fillers over covariants of IQ, age, and education by neurotypical controls (NC), first degree relatives (FDR), participants with (SZ+FTD), and without Formal Thought Disorder (SZ-FTD) in one-way analysis of variance.
**Table F.** Pairwise comparisons of neurotypical controls (NC), first degree relatives (FDR), participants with (SZ+FTD), and without Formal Thought Disorder (SZ-FTD) on the production of utterance-initial pauses in the Mann Whitney U Test.
**Table G.** Mean (M) and Standard Deviation (s.d.) for within-clause pauses with and without consideration of length.
**Table H.** Pairwise comparisons of neurotypical controls (NC), first degree relatives (FDR), participants with (SZ+FTD), and without Formal Thought Disorder (SZ-FTD) on the production of pauses before embedded clauses with and without length in Mann Whitney U Test.
**Table I.** Pairwise comparisons of neurotypical controls (NC), first degree relatives (FDR), participants with (SZ+FTD), and without formal thought disorder (SZ-FTD) on the production of fillers and their syntactic positions in the Mann Whitney U Test.
**Table J.** Mean (M) and Standard Deviation (s.d.) for within-clause fillers and fillers before embedded clauses.
**Figure A.** Average number of dysfluencies including both unfilled and filled pauses across neurotypical controls (NC), first degree relatives (FDR), participants with (SZ+FTD) and without thought disorder (SZ-FTD).
**Figure B.** Average of number of unfilled pauses across neurotypical controls (NC), first-degree relatives (FDR), participants with (SZ+FTD) and without thought disorder (SZ-FTD).
**Figure C.** Average number of utterance initial unfilled pauses across neurotypical controls (NC), first degree relatives (FDR), participants with (SZ+FTD) and without thought disorder (SZ-FTD).
**Figure D.** Average number of within-clause unfilled pauses across neurotypical controls (NC), first degree relatives (FDR), participants with (SZ+FTD) and without thought disorder (SZ-FTD).
**Figure E.** Average number of embedded clause unfilled pauses across neurotypical controls (NC), first degree relatives (FDR), participants with (SZ+FTD) and without thought disorder (SZ-FTD).
**Figure F.** Average number of filled pauses across neurotypical controls (NC), first degree relatives (FDR), participants with (SZ+FTD) and without thought disorder (SZ-FTD).
(DOCX)
