# Supplementary material for: Disturbing the rhythm of thought: Speech pausing patterns in schizophrenia, with and without formal thought disorder
Source: PLoS One. 2019 May 31;14(5):e0217404. doi: 10.1371/journal.pone.0217404 (PMC6544238; doi:10.1371/journal.pone.0217404)
Supplement: S1 File — Table A. Number of neurotypical controls (NC), first degree relatives (FDR), and participants with (SZ+FTD) and without formal thought disorder (SZ-FTD) for psychotropic medication. Table B. Speech dysfluency patterns. Table C. Statistical significance for possible between-group differences in speech dysfluencies. Table D. Pairwise comparisons of unfilled pauses/total utterances over covariants of IQ, age, and education by neurotypical controls (NC), first degree relatives (FDR), participants with FTD (SZ+FTD), and without FTD (SZ-FTD) in univariate analysis of variance. Table E. Pairwise comparisons of residuals of fillers over covariants of IQ, age, and education by neurotypical controls (NC), first degree relatives (FDR), participants with (SZ+FTD), and without Formal Thought Disorder (SZ-FTD) in one-way analysis of variance. Table F. Pairwise comparisons of neurotypical controls (NC), first degree relatives (FDR), participants with (SZ+FTD), and without Formal Thought Disorder (SZ-FTD) on the production of utterance-initial pauses in the Mann Whitney U Test. Table G. Mean (M) and Standard Deviation (s.d.) for within-clause pauses with and without consideration of length. Table H. Pairwise comparisons of neurotypical controls (NC), first degree relatives (FDR), participants with (SZ+FTD), and without Formal Thought Disorder (SZ-FTD) on the production of pauses before embedded clauses with and without length in Mann Whitney U Test. Table I. Pairwise comparisons of neurotypical controls (NC), first degree relatives (FDR), participants with (SZ+FTD), and without formal thought disorder (SZ-FTD) on the production of fillers and their syntactic positions in the Mann Whitney U Test. Table J. Mean (M) and Standard Deviation (s.d.) for within-clause fillers and fillers before embedded clauses. Figure A. Average number of dysfluencies including both unfilled and filled pauses across neurotypical controls (NC), first degree relatives (FDR), participants with (SZ+FTD) and w [file pone.0217404.s001.docx]

**Supplementary Materials**

| Types of medication | FDR | NC* | SZ+FTD | SZ-FTD |
| --- | --- | --- | --- | --- |
| Clozapine | ------- | ------- | 4 | 8 |
| Olanzapine | ------- | ------- | 6 | 2 |
| Amisulprid | ------- | ------- | 1 | 3 |
| Haloperidol | ------- | ------- | 2 | ------- |
| Risperidone | ------- | ------- | 1 | ------- |
| First-generation depot | ------- | ------- | 2 | 1 |
| Sulpiride | ------- | ------- | ------- | 1 |
| Aripiprazole | ------- | ------- | ------- | 3 |
| Quetiapine | ------- | ------- | ------- | 1 |
| SSRI | 1 | ------- | ------- | 1 |
| Venlafaxine | 1 | ------- | ------- | 1 |
| Mirtazepine | ------- | ------- | ------- | 1 |
| Temazepam | ------- | ------- | ------- | 1 |
| Zopiclone | ------- | ------- | 1 | 1 |
| Pregabalin | ------- | ------- | ------- | 1 |
| Sodium valproate | ------- | ------- | ------- | 2 |
| Thyroxine | 1 | ------- | ------- | ------- |
| Propranolol | 1 | ------- | ------- | ------- |
| Codeine | 1 | ------- | 1 | ------- |
| Selective serotonin reuptake inhibitor | ------- | ------- | 2 | ------- |
| Trazodone | ------- | ------- | 1 | ------- |
| Diazepam | ------- | ------- | 1 | ------- |
| Procyclidine | ------- | ------- | 3 | ------- |

*None were taking psychoactive drugs

**Table A.** Number of neurotypical controls (NC), first degree relatives (FDR), and participants with (SZ+FTD) and without formal thought disorder (SZ-FTD) for psychotropic medication.

| Dysfluency types | Syntactic positions | Example | Explanation |
| --- | --- | --- | --- |
|  | Utterance-initial | **(PAUSE<1sec)** He phoned his friend | Unfilled pause is at the beginning of the utterance. |
| Unfilled pauses | Within-clause pauses | He phoned **(PAUSE <1sec)** his friend | Unfilled pause is between a verb and an NP. |
|  | Pauses before embedded clauses | He phoned his friend **(PAUSE <1sec)** whom he has not seen since the graduation. | Unfilled pause before an embedded clause (in this case a relative). |
|  |  |  |  |
| Filled pauses | Utterance-initial filled pause | **(FILLER)** He phoned his friend. | Filled pause is at the beginning of the utterance. |
|  | Within-clause filled pause | London is **(FILLER)** my favourite capital city. | Filled pause is within a clause. |
|  | Filled pauses before embedded clauses | He phoned his friend **(FILLER)** whom he has not seen since the graduation. |  |

**Table B.** Speech dysfluency patterns

| Speech dysfluencies | Ratios | Model Type | Statistics | p | η^2^ |
| --- | --- | --- | --- | --- | --- |
|  | Dysfluency with unfilled and filled/total utterances | Univariate-GLM | F (3,59) = .742 | .531 | None |
| Unfilled pauses | Unfilled pauses/total utterances | One-way analysis of covariance (ANCOVA) | F (3,59) = 1.509 | .223 | None |
| Fillers | Fillers/total utterances | Rank analysis of covariance (Quade) & one-way analysis of variance | F (3,12009) = 5.660 | .002^1^ | .23 |
| Syntactic positions of | Utterance-initial unfilled pauses/ total utterances | Kruskal-Wallis | H (3) = 10.249 | .017* | .13 |
| unfilled pauses | Within-clause unfilled pauses excluding embedded clauses/total clauses^2^ | Kruskal-Wallis | H (3) = 2.230 | .526 | None |
|  | Unfilled pauses before embedded clauses/total embedded clauses^3^ | Kruskal-Wallis | H (3) = 13.917 | .003* | .20 |
| Duration of unfilled pauses | Utterance-initial pauses less than 1 sec/total utterances | Kruskal-Wallis | H (3) = 3.750 | .289 | None |
|  | Utterance-initial pauses between 1 and 3 secs/total utterances | Kruskal-Wallis | H (3) = 13.250 | .004* | .12 |
|  | Within-clause pauses excluding embedded clauses less than 1 sec /total clauses | Kruskal-Wallis | H (3) = .682 | .877 | None |
|  | Within-clause pauses excluding embedded clause between 1 and 3 sec /total clauses | Kruskal-Wallis | H (3) = 4.596 | .204 | None |
|  | Unfilled pauses before embedded clauses less than 1 sec / total embedded clauses^3^ | Kruskal-Wallis | H (3) = 5.747 | .125 | None |
|  | Unfilled pauses before embedded clauses between 1 and 3 sec/ total embedded clauses | Kruskal-Wallis | H (3) = 10.079 | .018* | .13 |
| Syntactic positions of | Utterance-initial fillers/ total utterances | Kruskal-Wallis | H (3) = 8.239 | .040* | .10 |
| filled pauses | Within-clause fillers/total clauses | Kruskal-Wallis | H (3) = 1.002 | .801 | None |
|  | Fillers before embedded clauses/ total embedded clause | Kruskal-Wallis | H (3) = .720 | .869 | None |

^1^Mean difference significant at .05 level. The co-variants are IQ, age and education. ^2^The total number of clauses includes the number of utterances and embedded clauses. ^3^The total number of embedded clauses does not include the total number of utterances.

[dx.doi.org/10.17504/protocols.io.zqrf5v6](http://dx.doi.org/10.17504/protocols.io.zqrf5v6)

**Table C.** Statistical significance for possible between-group differences in speech dysfluencies.

|  |  |  |  |  |  |  |  |  |
| --- | --- | --- | --- | --- | --- | --- | --- | --- |
|  |  |  |  |  |  |  | 95% CI | |
| Linguistic Variable | Group | Mean (SD) | Comparisons | Mean difference | SE | p | Lower Bound | Upper Bound |
| Unfilled pauses/total utterances | NC | .83 (.33) | NC vs. SZ-FTD | -.421 | .223 | .387 | -.1.03 | .190 |
|  | FDR | .90 (.30) | NC vs. SZ+FTD | -.128 | .246 | 1.00 | -.801 | .545 |
|  | SZ-FTD | 1.30 (.63) | NC vs. FDR | -.020 | .198 | 1.00 | -.562 | .522 |
|  | SZ+ FTD | 1.10 (.74) | FDR vs. SZ- FTD | -.401 | .217 | .423 | -.997 | .195 |
|  | ------- | ------- | FDR vs. SZ+FTD | -.109 | .231 | 1.00 | -.743 | .526 |
|  | -------- | ------- | SZ+ FTD vs. SZ-FTD | -.293 | .221 | 1.00 | -.899 | .314 |

* The mean difference is significant at the .05 level.

**Table D.** Pairwise comparisons of unfilled pauses/total utterances over covariants of IQ, age, and education by neurotypical controls (NC), first degree relatives (FDR), participants with FTD (SZ+FTD), and without FTD (SZ-FTD) in univariate analysis of variance.

|  |  | Comparisons | Mean Difference (I-J) | Std. Error | Sig. | 95% CI | |
| --- | --- | --- | --- | --- | --- | --- | --- |
| Group | Mean (s.d) |  |  |  |  | Lower Bound | Upper Bound |
| NC | 11 (3.78) | NC vs. SZ-FTD | 12.912 | 5.347 | .114 | -1.714 | 27.538 |
| FDR | -11 (3.78) | NC vs. SZ+ FTD | 7.776 | 5.347 | .909 | -6.850 | 22.402 |
| SZ+FTD | -2.384 (3.78) | NC vs. FDR | 21.422^*^ | 5.347 | .001* | 6.796 | 36.048 |
| SZ-FTD | 2.752 (3.78) | FDR vs. SZ-FTD | -8.510 | 5.347 | .703 | -23.136 | 6.116 |
|  |  | FDR vs. SZ+ FTD | -13.646 | 5.347 | .081 | -28.272 | .980 |
|  |  | SZ-FTD vs. SZ+ FTD | -5.136 | 5.347 | 1.000 | -19.762 | 9.490 |

**Table E.** Pairwise comparisons of residuals of fillers over covariants of IQ, age, and education by neurotypical controls (NC), first degree relatives (FDR), participants with (SZ+FTD), and without Formal Thought Disorder (SZ-FTD) in one-way analysis of variance.

| Linguistic Variables | Group | Mean  (s.d.) | Comparisons | U | Z | Sig.  (2-tailed) | Effect size (***η^2^***) | Bonferroni correction |
| --- | --- | --- | --- | --- | --- | --- | --- | --- |
|  | NC | .42 (.20) | NC vs. SZ- FTD | 183.500 | 2.945 | .002* | .29 | <.008* |
| Utterance-initial pauses/total utterances  (disregarding the length of pauses) | FDR | .49 (.22) | NC vs. SZ+FTD | 157.00 | 1.846 | .067 | None | >.008 |
|  | SZ-FTD | .76 (36) | NC vs. FDR | 128.00 | .643 | .539 | None | >.008 |
|  | SZ+FTD | .63 (38) | FDR vs. SZ- FTD | 170.500 | 2.406 | .015 | .19 | >.008 |
|  |  |  | FDR vs. SZ+FTD | 140.500 | 1.162 | .250 | None | >.008 |
|  |  |  | SZ-FTD vs.  SZ+FTD | 98.000 | -.601 | .548 | None | >.008 |
| Utterance-initial pauses less than one sec/total utterances | NC | .32 (.19) | ----------------- | ------------ | --------- | --------- | --------- | --------- |
|  | FDR | .33 (.20) | ----------------- | ------------ | --------- | --------- | --------- | --------- |
|  | SZ-FTD | .43 (.30) | ----------------- | ------------ | --------- | --------- | --------- | --------- |
|  | SZ+FTD | .24 (.19) | ----------------- | ------------ | --------- | --------- | --------- | --------- |
| Utterance-initial pauses between one | NC | .10 (.07) | NC vs. SZ- FTD | 175.500 | 2.618 | .008* | .22 | =.008* |
| and three secs/total utterances | FDR | .16 (.15) | NC vs. SZ+FTD | 189.000 | 3.178 | .001* | .33 | <.008* |
|  | SZ-FTD | .33 (.28) | NC vs. FDR | 133.500 | .021 | .389 | None | >.008 |
|  | SZ+FTD | .39 (.32) | FDR vs. SZ- FTD | 150.500 | 1.579 | .116 | None | >.008 |
|  |  |  | FDR vs. SZ+FTD | 161.500 | 2.476 | .021 | .14 | >.008 |
|  |  |  | SZ-FTD vs.  SZ+FTD | 132.500 | .830 | .412 | None | >.008 |

**Table F.** Pairwise comparisons of neurotypical controls (NC), first degree relatives (FDR), participants with (SZ+FTD), and without Formal Thought Disorder (SZ-FTD) on the production of utterance-initial pauses in the Mann Whitney U Test

| Linguistic Variables | Group | Mean  (s.d.) |
| --- | --- | --- |
| Within-clause pauses excluding subordinate clauses/total clauses^1^  (disregarding the length of pauses) | NC | .23 (.14) |
|  | FDR | .22 (.23) |
|  | SZ-FTD | .27 (.18) |
|  | SZ+FTD | .31 (.34) |
| Within-clause pauses excluding subordinate clauses  less than one sec/total clauses | NC | .20 (.13) |
|  | FDR | .21 (.23) |
|  | SZ-FTD | .22 (.17) |
|  | SZ+FTD | .23 (.26) |
| Within-clause pauses excluding | NC | .03 (.05) |
| subordinate clauses between one and three secs/total clauses | FDR | .01 (.02) |
|  | SZ-FTD | .05 (.06) |
|  | SZ+FTD | .08 (.12) |

^1^The total number of clauses includes the number of utterances and embedded clauses.

**Table G.** Mean (M) and Standard Deviation (s.d.) for within-clause pauses with and without consideration of length

| Linguistic Variables | Group | Mean  (s.d.) | Comparisons | U | Z | Sig.  (2‐tailed) | Effect size (***η^2^***) | Bonferroni correction |
| --- | --- | --- | --- | --- | --- | --- | --- | --- |
|  | NC | .22 (.13) | NC vs. SZ- FTD | 200.000 | 3.637 | .001* | .45 | <.008* |
| Pauses before embedded clauses/ total embedded clauses (disregarding the length of pauses) | FDR | .37 (.34) | NC vs. SZ+FTD | 108.000 | -.188 | 870 | None | >.008 |
|  | SZ-FTD | .50 (.21) | NC vs. FDR | 143.000 | 1.268 | 217 | None | >.008 |
|  | SZ+FTD | .23 (.22) | FDR vs. SZ- FTD | 158.500 | 1.914 | .056 | None | >.008 |
|  |  |  | FDR vs. SZ+FTD | 83.500 | -1.216 | .233 | None | >.008 |
|  |  |  | SZ-FTD vs.  SZ+FTD | 45.000 | -2.815 | .004* | .18 | <.008* |
| Pauses before embedded clauses less | NC | .20 (.12) | ----------------- | ------------- | ----------- | ----------- | ----------- | ----------- |
| than one sec/total embedded clauses | FDR | .30 (.32) | ----------------- | ------------- | ----------- | ----------- | ----------- | ----------- |
|  | SZ-FTD | .33 (.19) | ----------------- | ------------- | ----------- | ----------- | ----------- | ----------- |
|  | SZ+FTD | .18 (.19) | ----------------- | ------------- | ----------- | ----------- | ----------- | ----------- |
| Pauses before embedded clauses | NC | .02 (.04) | NC vs. SZ- FTD | 177.500 | 2.928 | .006* | .024 | <.008* |
| between one and three secs/total embedded clauses | FDR | .07 (.11) | NC vs. SZ+FTD | 122.500 | .512 | .683 | None | >.008 |
|  | SZ-FTD | .17 (.15) | NC vs. FDR | 137.500 | 1.236 | .305 | None | >.008 |
|  | SZ+FTD | .05 (.09) | FDR vs. SZ- FTD | 153.500 | 1.796 | .089 | None | >.008 |
|  |  |  | FDR vs. SZ+FTD | 99.500 | -.624 | .595 | None | >.008 |
|  |  |  | SZ-FTD vs.  SZ+FTD | 63.000 | -2.195 | .041 | None | >.008 |

**Table H.** Pairwise comparisons of neurotypical controls (NC), first degree relatives (FDR), participants with (SZ+FTD), and without Formal Thought Disorder (SZ-FTD) on the production of pauses before embedded clauses with and without length in Mann Whitney U Test

| Linguistic Variables | Group | Mean  (s.d.) | Comparisons | U | Z | Sig.  (2‐tailed) | Effect size (***η^2^***) | Bonferroni correction |
| --- | --- | --- | --- | --- | --- | --- | --- | --- |
| Utterance-initial fillers | NC | .32 (.21) | NC vs. SZ- FTD | 168.000 | 2.308 | .022 | None | >.008 |
| / total utterances | FDR | .14 (.14) | NC vs. SZ+FTD | 157.500 | 1.868 | .062 | None | >.008 |
|  | SZ-FTD | .16 (.20) | NC vs. FDR | 176.000 | 2.638 | .008* | .23 | =.008* |
|  | SZ+FTD | .20 (.20) | FDR vs. SZ- FTD | 116.000 | .147 | .900 | None | >.008 |
|  |  |  | FDR vs. SZ+FTD | 101.000 | -.480 | -.906 | None | >.008 |
|  |  |  | SZ-FTD vs.  SZ+FTD | 125.500 | .298 | .1000 | None | >.008 |

**Table I.** Pairwise comparisons of neurotypical controls (NC), first degree relatives (FDR), participants with (SZ+FTD), and without formal thought disorder (SZ-FTD) on the production of fillers and their syntactic positions in the Mann Whitney U Test.

| Linguistic Variables | Group | Mean  (s.d.) |
| --- | --- | --- |
| Within-clause fillers/total clauses | NC | .77 (1.07) |
|  | FDR | .33 (.48) |
|  | SZ-FTD | .44 (.65) |
|  | SZ+FTD | .77 (1.06) |
| Fillers before embedded clauses/total embedded clauses | NC | .049 (.099) |
|  | FDR | .020 (.047) |
|  | SZ-FTD | .043 (.091) |
|  | SZ+FTD | .052 (.090) |

**Table J.** Mean (M) and Standard Deviation (s.d.) for within-clause fillers and fillers before embedded clauses


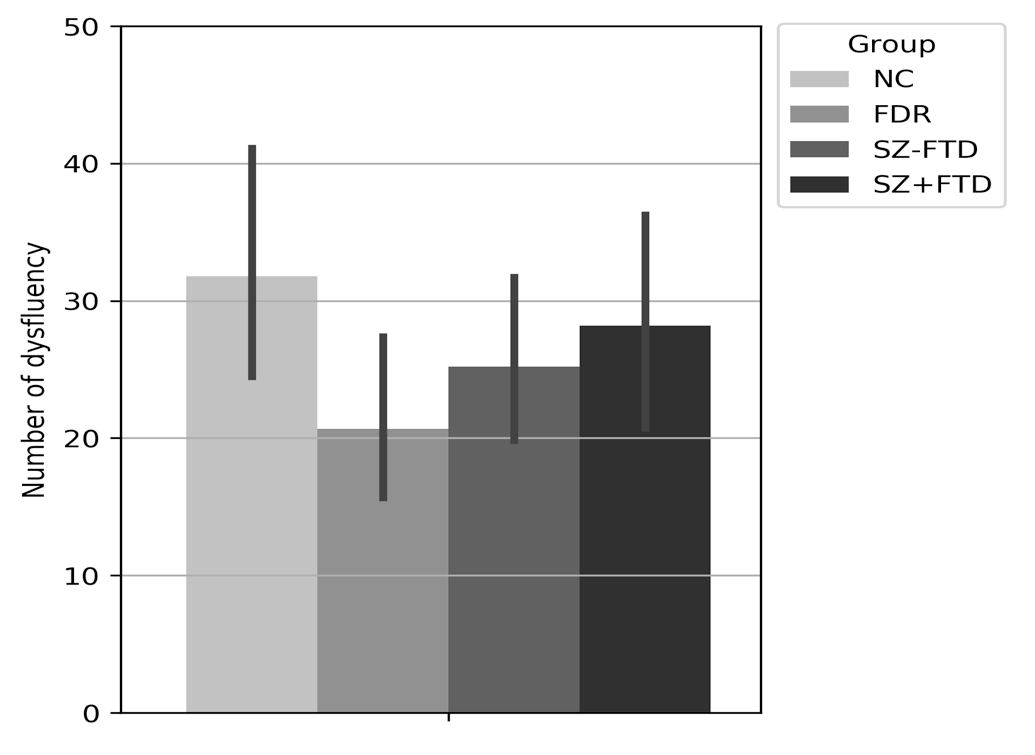


**Figure A**. Average number of dysfluencies including both unfilled and filled pauses across neurotypical controls (NC), first degree relatives (FDR), participants with (SZ+FTD) and without thought disorder (SZ-FTD).


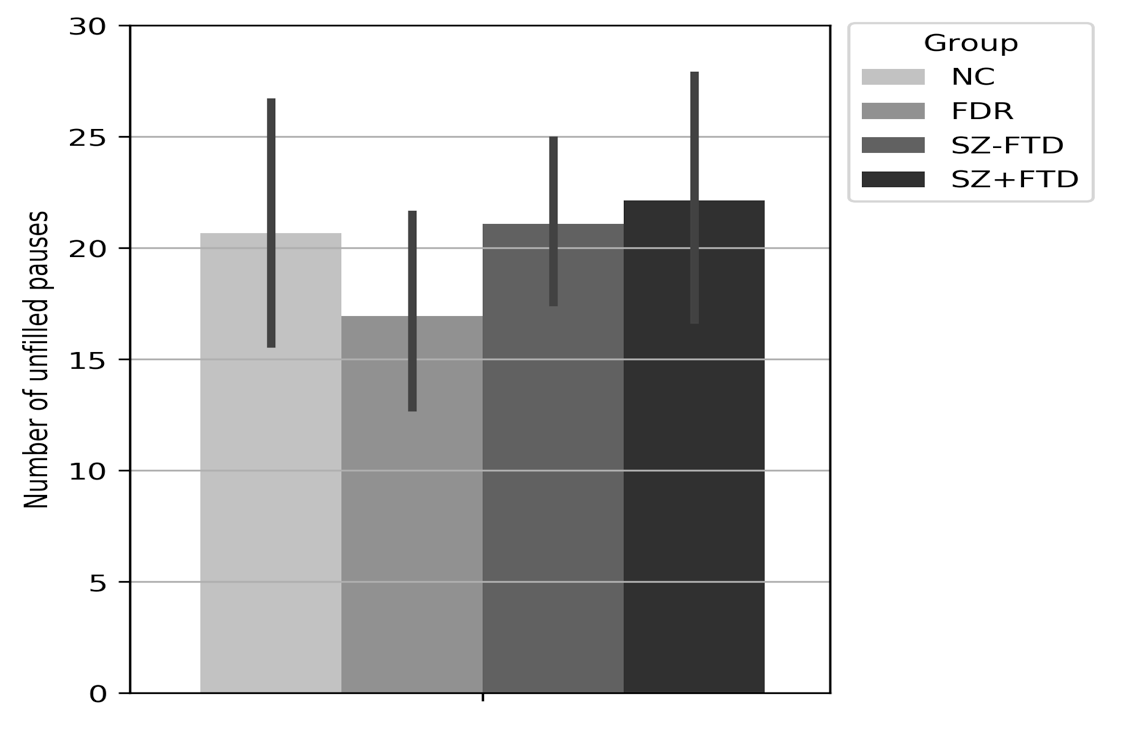


**Figure B**. Average of number of unfilled pauses across neurotypical controls (NC), first-degree relatives (FDR), participants with (SZ+FTD) and without thought disorder (SZ-FTD).


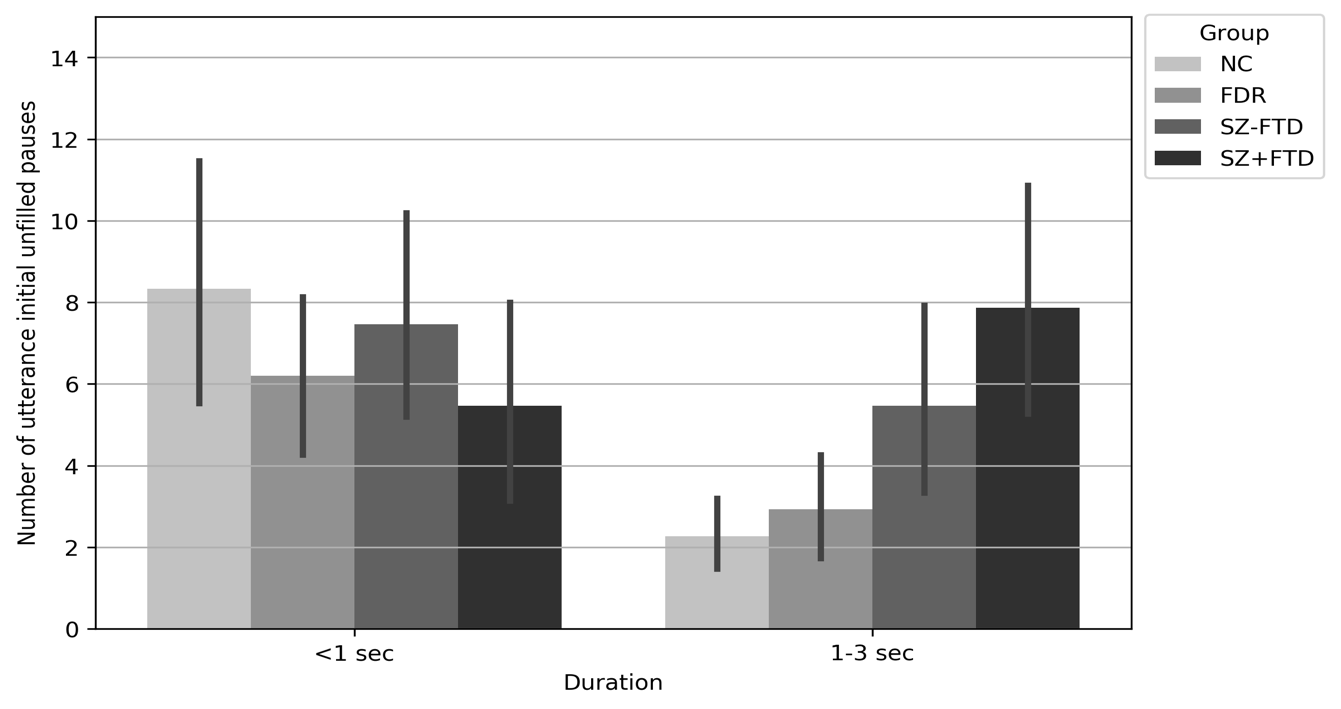


**Figure C**. Average number of utterance initial unfilled pauses across neurotypical controls (NC), first degree relatives (FDR), participants with (SZ+FTD) and without thought disorder (SZ-FTD).


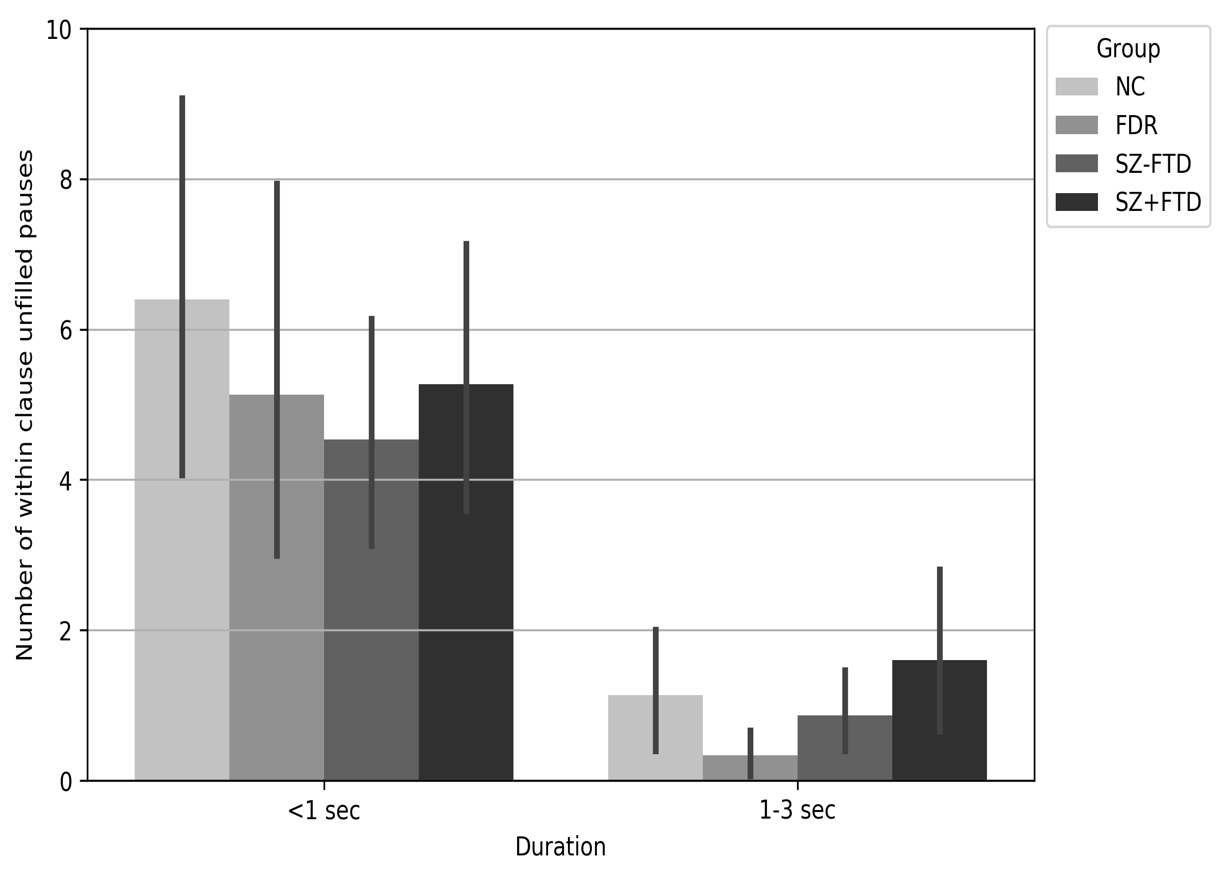


**Figure D**. Average number of within-clause unfilled pauses across neurotypical controls (NC), first degree relatives (FDR), participants with (SZ+FTD) and without thought disorder (SZ-FTD).


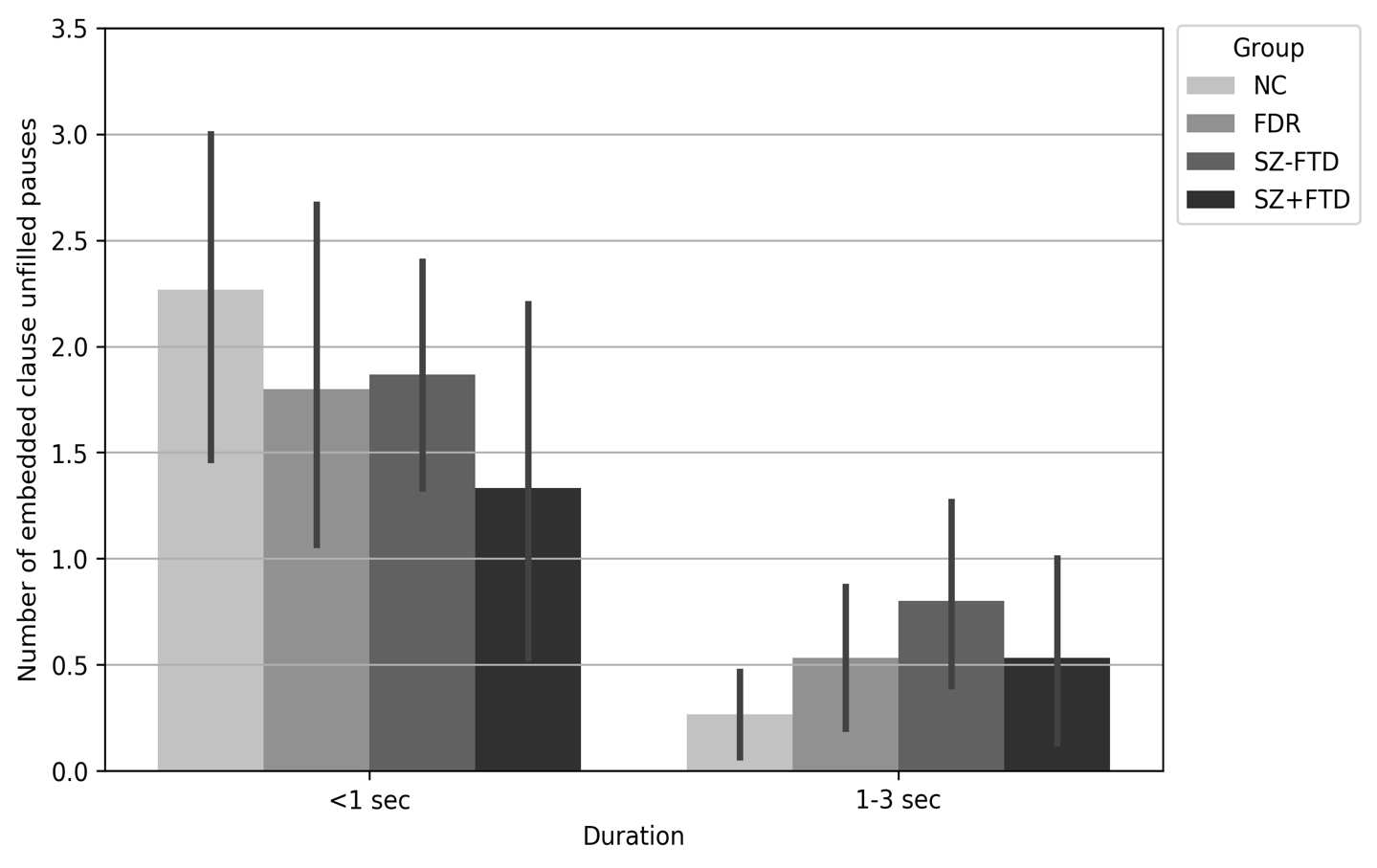


**Figure E**. Average number of embedded clause unfilled pauses across neurotypical controls (NC), first degree relatives (FDR), participants with (SZ+FTD) and without thought disorder (SZ-FTD).


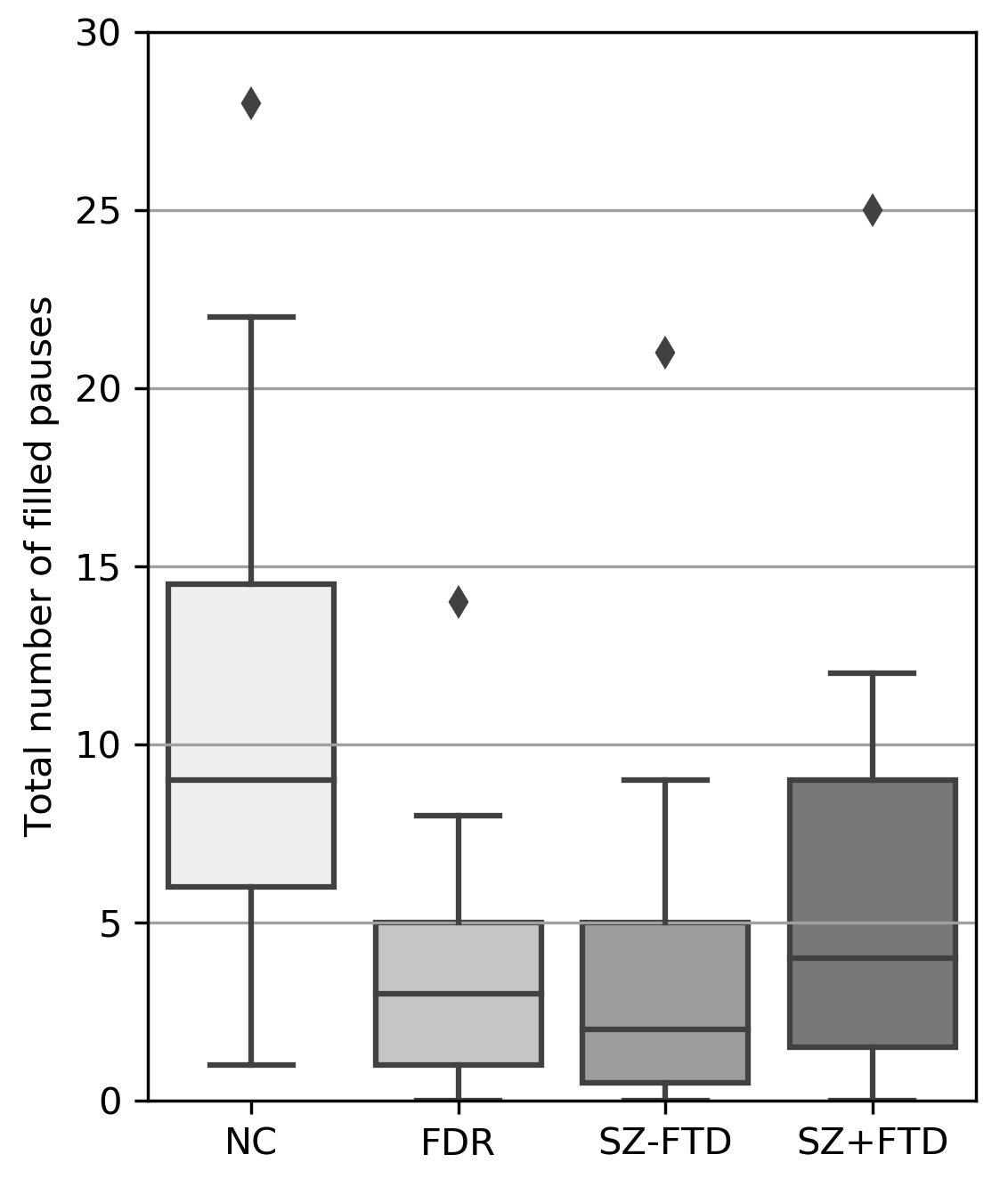


**Figure F**. Average number of filled pauses across neurotypical controls (NC), first degree relatives (FDR), participants with (SZ+FTD) and without thought disorder (SZ-FTD).
